# 

**Published:** 1932

**Authors:** 


### The Medical Library of the University of Bristol,

WITH WHICH IS INCORPORATED

**The Library of the Bristol Medico-Chirurgical Society.**

*The following donations have been received since the publication of the list in March,* 1932.

June, 1932.

| | |
|---|---|
| American Laryngological Association (1) .. | 1 volume. |
| American Laryngological, Rhinological and Otological Soc. (2) .. .. .. .. | 1 ,, |
| Groves, Professor E. W. Hey (3) .. .. | 6 volumes. |
| Kruse, Fritz (4) .. .. .. .. .. | 1 volume. |
| Smith, W. Alexander, M.A., M.B. (5) .. .. | ,, |
| Walker, Cyril H., B.A., M.B., F.R.C.S. (6) .. | 15 volumes. |
| Wilmer Institution (7) .. .. .. .. | 1 volume. |

Unbound periodicals have also been received from Professor E. W. Hey Groves and Dr. Carey F. Coombs.

### THE ONE HUNDRED AND FORTIETH LIST OF BOOKS.

The figures in round brackets refer to the figures after the names of the donors. The books to which no such figures are attached have either been bought from the Library Fund or received through the *Journal.*

| | | | |
|---|---|---|---|
| **Allison, N., and Ghormley, R. K.** | *Diagnosis in Joint Disease* | | 1931 |
| **Bailey, Hamilton** | *Emergency Surgery* | Vol. 2 | 1931 |
| **Bertwistle, A. P.** | *Atlas of Radiographs* | 4th Ed. (3) | 1932 |
| *Birth Control and Public Health* | | | 1932 |
| **Black, G.** | *Eyesight and How to Care for It* | (6) | |
| **Christie, H. K.** | *Technique and Results of Grafting Skin* | | 1930 |
| **Coates, V., and Delicati, L.** | *Rheumatoid Arthritis* | | 1931 |
| **Cornioley, C. E.** | *L'Ostéosynthèse des os longs* | (3) | 1931 |
| **Crookshank, F. G.** | *Individual Sexual Problems* | | 1931 |
| **De Vilhena, H.** | *A expressão física da Cólera* | 2nd Ed. | 1931 |
| **Ewart, E. D.** | *A Guide to Anatomy* | 3rd Ed. | 1932 |
| **Fifield, L. R.** | *Minor Surgery* | 2nd Ed. | 1931 |
| **Forsdike, S.** | *Textbook of Gynæcology* | | 1932 |
| **Gant, F. J.** | *Guide to the Examinations of the Royal College of Surgeons of England* | (6) | 1881 |
| **Greene, C. L. G.** | *Medical Diagnosis for the Student and Practitioner* | (5) | [1917] |
| **Guthrie, A. C.** | *Research Work on the Pneumococci and their Enzymes* | | 1932 |
| **Gye, W. E., and Purdy, W. J.** | *Cause of Cancer* | | [1931] |
| **Haret, G., Dariaux, A., et Quénu, J.** | *Atlas de radiographie Osseuse 3 Tomes* | (3) | 1931 |
| **Hill, L.** | *Recent Advances in Physiology and Bio-Chemistry* | (6) | 1908 |
| **Hillier, W. T.** | *A Theory of the Formation of Animals* | | 1932 |
| **Howe, E. G.** | *Motives and Mechanisms of the Mind* | | 1931 |
| **Hutton, J. E.** | *The Hygiene of Marriage* | 3rd Ed. | 1932 |
| **Huxley, J. H.** | *Lessons in Elementary Physiology* | (6) | 1888 |
| **Isenthal, A. W., and Ward, A. S.** | *Practical Radiography* | (6) | 1898 |
| **Jackson, J. H.** | *Selected Writings of* | Vol. 2 | 1932 |
| **Javal, E.** | *Blind Man's World* | (6) | 1904 |
| **Kirchner, M.** | *Operationslehre II* | (3) | 1932 |
| **Knapp, A.** | *Medical Ophthalmology* | (6) | 1918 |
| **Knies** | *Relations of Diseases of the Eye to General Diseases* | (6) | 1895 |
| **Kruse, Fritz** | *Die Lösung des Problems der echten Epilepsie* | (4) | |
| *Lancet Commission on Nursing—Final Report* | | | 1932 |
| **Lawrence, R. D.** | *Diabetic Life* | 6th Ed. | 1931 |
| **Le Conte, J.** | *Sight* | (6) | 1881 |
| **Lohmann, (W.)** | *Disturbances of the Visual Functions* | (6) | 1913 |
| **Mollison, W. M.** | *Acute Otitis Media* | | 1932 |
| **Parry, L. A.** | *Criminal Abortion* | | 1932 |
| *Pharmacopœia of the London Hospital* | | (6) | 1882 |

**Porter, C., and Fenton, J.** *Sanitary Law in Question and Answer* 3rd Ed. 1932
**Richardson, B. W.** .. *Biological Experimentation* .. .. .. (6) 1896
**Schnek, F.** .. .. *Technique of the Non-padded Plaster Cast* .. 1932
**Short, A. R.** .. .. *Index of Prognosis* .. .. .. .. .. 4th Ed. 1932
**Stephens, G. A.** .. *Heart and Spleen in Health and Disease* .. 1932
**Stubbs, S. G. B., and Bligh, E. W.** *Sixty Centuries of Health and Physick* .. .. .. .. .. .. .. .. .. .. [ ]
**Symes, J. O.** .. .. *Short History of the Bristol General Hospital* 1932
**Thompson, C. J. S.** .. *Quacks of Old London* .. .. .. .. .. (6) 1928
**Turnbull, A.** .. .. *Treatment of Diseases of the Eye* .. (6) 1843
**Usland, O.** .. .. .. *Kirurgiske Pankreassykdommer* .. .. .. 1932
**Wallace, O.** .. .. *Wayfaring in a Splint* .. .. .. .. .. .. 1931

TRANSACTIONS, REPORTS, JOURNALS, ETC.

American Laryngological Association, Transactions 53rd Annual Meeting .. .. .. .. .. .. .. .. .. .. .. .. .. .. (1) 1931
American Laryngological, Rhinological and Otological Society, Transactions 37th Annual Meeting .. .. .. .. .. .. .. .. (2) 1931
Medical Annual .. .. .. .. .. .. .. .. .. .. .. .. .. .. .. .. .. 1932
Quarterly Cumulative Index Medicus. Vol. 10 (1931) .. .. .. 1932
Surgical Clinics of North America. Vol. 12, No. 2 .. .. .. .. 1932
Universities Year Book .. .. .. .. .. .. .. .. .. .. .. .. .. .. 1931
Wilmer Institution, Collected Reprints. Vol. II.. .. .. .. (7) 1932

### Local Medical Notes

EXAMINATION RESULTS.

**University of Bristol.**—Students of the University have recently passed the following examinations :—

M.B., CH.B.—*First Examination. Pass :* Daphne V. Dennis, J. P. M. Forde, H. D. T. Gawn, F. J. W. Hooper, C. R. G. Howard, C. B. Jones, Jane Mackintosh, Margaret E. Morgan, G. L. Page, A. N. H. Peach, P. B. Ryan, R. J. K. Tallack, R. M. W. Travers, T. R. de V. Vallentin. *In Physics and Chemistry* (*Completing Requirements*) *:* Margaret H. Davies. *In Physics and Botany* (*Biology*) *Completing Requirements :* N. R. Matheson.

M.B., CH.B. (*Part I.*)—*Second Examination. Pass : In Organic Chemistry* (*Completing Examination*) *:* Margaret E. Morgan.

M.B., CH.B. (*Part I.*).—*Final Examination. Pass :* Margaret A. W. Hawkes (with distinction in Materia Medica, Pharmacy, Pharmacology and Therapeutics), A. V. House.

M.B., CH.B. (*Part II.*)—*Second Examination. Pass :* J. W. E. Snawdon.

M.B., CH.B. (*Part II.*).—*Final Examination. Pass :* (with First Class Honours), A. L. Eyre-Brook (with distinction in Surgery, Obstetrics and Pathology). *Pass :* (with Second Class Honours), O. E. L Sampson. *Pass :* K. P. Beaubrun, G. T. Bevir, R. G. C. G. Carlson, A. McD. Davies, H. F. M. Finzel, F. E. Fletcher, J. R. Gibbs (with Distinction in Forensic Medicine and Toxicology), Frances N. Salisbury.

M.D.—P. Phillips.

B.D.S.—*First Examination. Pass :* A. N. Brimelow, C. C. B. Plumley.

PRELIMINARY SCIENCE EXAMINATION FOR DENTAL STUDENTS (L.D.S.).—*Pass : In Chemistry (Completing Examination) :* D. E. Royal. *In Chemistry and Physics only :* R. A. Hilton.

PRELIMINARY SCIENCE EXAMINATION FOR DENTAL STUDENTS.—*Pass : In Biology only (Completing Examination) :* R. A. Hilton. *In Biology only :* J. F. Durie, H. D. Williams.

L.D.S. — *First Professional Examination. Pass : In Anatomy and Physiology (Completing Examination) :* Frances M. Orton, L. E. Scull. *In Anatomy and Physiology only :* B. E. Ffrench, D. E. Royal. *In Dental Anatomy only :* L. K. A. S. Oxley. *Second Professional Examination. Pass :* J. Hewitt. *In Dental Materia Medica (Completing Examination) :* J. H. G. Smith.

L.D.S. — *Second Professional Examination. Pass :* J. Hewitt. *In Dental Materia Medica (Completing Examination) :* J. H. G. Smith.

L.D.S.—*Final Professional Examination. Pass :* Helena C. L. Blinkworth, W. V. Whiteford.

**Conjoint Board.** — M.R.C.S., L.R.C.P. — *In Chemistry :* A. L. T. Beddoe. *In Anatomy :* W. H. Hayes. *In Medicine :* Enid A. Taylour, A. M. Davies.* *In Medicine and Surgery :* O. E. L. Sampson,* A. L. Eyre-Brook,* H. F. M. Finzel,* Frances N. Salisbury,* F. E. Fletcher. *In Surgery :* G. T. Bevir, J. L. S. James. *In Midwifery :* Annie M. B. Walker, J. F. H. Eagles, E. O. Walker.

* Qualified.

L.D.S., R.C.S.—*First Professional Examination. Pass: In Dental Mechanics and Dental Metallurgy:* W. B. Lewis. *In Dental Metallurgy and Dental Anatomy and Physiology:* J. H. G. Morris. *In Dental Metallurgy:* W. White. *In Anatomy and Physiology:* L. Jonathan. *In Dental Anatomy and Physiology:* Beryl M. Davies, N. G. Harrison, D. Gent, J. D. Rees.

L.D.S., R.C.S.—*Final Examination. Part I.:* F. S. Bromley, J. S. Cochrane, R. H. Green, S. A. McCandlish, P. J. F. Shepherd, H. W. South, W. N. Trays.

**Society of Apothecaries.** — L.S.A. — *Pre-Medical and Primary Examinations. Pass (in Physiology only):* K. P. Pauli. *Final Examination. Pass (in Medicine only):* (Mrs.) Beryl Busby.

---

**Bristol General Hospital.**—The following appointments have been made :—Consulting Surgeon : E. W. Hey Groves, M.S., F.R.C.S. Consulting Physician Accoucheur : D. C. Rayner, F.R.C.S. Surgeon : D. G. C. Tasker, M.S., F.R.C.S. Physician Accoucheur : H. J. Drew Smythe, M.C., M.S., F.R.C.S., M.M. Assistant Surgeon : G. M. Fitz-Gibbon, M.D., F.R.C.S. Assistant Physician Accoucheur : F. J. Hector, M.D., F.R.C.S.

**A British Medical Association Prize,** awarded for the best essay, contributed by a student of the Faculty of Medicine in any one of the following Universities : Birmingham, Bristol, Durham, Leeds, Liverpool, Manchester, Sheffield and the Welsh National School of Medicine, on "The Importance of the Condition of the Teeth to the Medical Practitioner," has been gained by Mr. H. E. Pearse (Bristol).

**Long Fox Memorial Lecture.**—Mr. A. J. M. Wright, F.R.C.S., has been appointed Long Fox Lecturer for 1932.

**Colston Medical Research Fellowship.**—A Fellowship of £200 for the year ending 31st August, 1933, has been awarded to Dr. Bruce Perry for "An Investigation into the Ætiology of Coronary Occlusion."

### Publications Received

From BAILLIÈRE, TINDALL & COX:

*Baillière's Synthetic Anatomy.* By J. E. CHEESMAN. Price 42s.

From JOHN BALE, SONS AND DANIELSSON LTD.:

*The Discharging Ear.* By A. G. WELLS, M.S., M.B. Price 2s 6d.

From W. GREEN AND SON LTD.:

*An Introduction to Dermatology.* 9th Edition. By NORMAN WALKER, M.D., and G. H. PERCIVAL, M.D. Price 20s.

From H. K. LEWIS AND CO., LTD.:

*Sanitary Law in Question and Answer.* 3rd Edition. By CHARLES PORTER, M.D., B.Sc., M.R.C.P., and JAMES FENTON, M.D., D.P.H. Price 7s. 6d.
*A Guide to Anatomy.* 3rd Edition. By E. D. EWART. Price 12s. 6d.
*A Guide to General Practice.* By A. H. DOUTHWAITE, M.D. Price 4s. 6d.
*Cancer: Civilization: Degeneration.* By J. COPE. Price 15s.
*A Short Practice of Surgery.* Vol. I. By HAMILTON BAILEY and R. J. MCNEILL LOVE, M.S. Price 20s.
*Choosing a Wife and other Essays.* By E. G. D. DRURY, M.D., B.S., D.P.H. Price 8s. 6d.

From MACMILLAN AND CO.:

*Empire Review.* No. 373. February 1932. Price 2s.

From WILHELM MAUDRICH:

*The Technique of the Non-Padded Plaster Cast.* By FRITZ SCHNEK, M.D. Price $5.
*Plastic Surgery of the Nose, Ear and Face.* By VICTOR FRÜHWALD. Price $4.

From OXFORD UNIVERSITY PRESS:

*The Use of Lipiodol in Diagnosis and Treatment.* By J. A. SICARD and J. FORESTIER. Price 16s.

From SOCIETY FOR THE PROVISION OF BIRTH CONTROL CLINICS:

*Birth Control and Public Health.* Price 1s.

From JOHN WRIGHT AND SONS LTD.:

*A Short History of the Bristol General Hospital.* By J. ODERY SYMES, M.D.
*A New Theory of Cancer and its Treatment.* By C. F. MARSHALL, M.Sc., M.D. Price 3s. 6d.
*British Journal of Surgery.* Volume XIX. No. 76. Price 12s. 6d.
*Medical Annual,* 1932. Price 20s.

